# Supplementary material for: MetaRibo-Seq measures translation in microbiomes
Source: Nat Commun. 2020 Jun 29;11:3268. doi: 10.1038/s41467-020-17081-z (PMC7324362; doi:10.1038/s41467-020-17081-z)
Supplement: Supplementary file 10 — Supplementary Data 7 [file 41467_2020_17081_MOESM10_ESM.zip › File2/Confidence_High_Taxonomy/86005_out.krona.html]

Javascript must be enabled to view this page.

members
magnitude
magnitudeUnassigned
count
unassigned
taxon
rank

86005\_out

3

3
superkingdom
2

3
phylum
1239

91061
class
2

1385
2
order

family
1
186820

1637
1
genus

1639

SRS017821\_contig\_number\_18603
1
species

186817
1
family

genus
1
1386

species group
1

SRS147272\_contig\_number\_contig-100\_5196.5197
86661

186801
class
1

1
order

SRS014613\_contig\_number\_22281
186802
